# Progress on Salt Tolerance in *Brassica napus*

**DOI:** 10.3390/plants13141990

**Published:** 2024-07-21

**Authors:** Rui Dai, Na Zhan, Rudan Geng, Kun Xu, Xiangchun Zhou, Lixia Li, Guixin Yan, Fanglin Zhou, Guangqin Cai

**Affiliations:** 1 Key Laboratory of Biology and Genetic Improvement of Oil Crops, Ministry of Agriculture and Rural Affairs, Oil Crops Research Institute, Chinese Academy of Agricultural Sciences, Wuhan 430062, China; d18846916404@163.com (R.D.); zhanna2000@163.com (N.Z.); gengrudan@caas.cn (R.G.); xk1919@163.com (K.X.); zhouxiangchun@caas.cn (X.Z.); linen19880915@163.com (L.L.); ygx_928@126.com (G.Y.); 2 Hubei Hongshan Laboratory, Wuhan 430070, China

**Keywords:** *Brassica napus*, salt tolerance, molecular mechanism, physiological and phenotypic indexes, exogenous substances

## Abstract

In China, saline–alkali lands constitute 5.01% of the total land area, having a significant impact on both domestic and international food production. Rapeseed (*Brassica napus* L.), as one of the most important oilseed crops in China, has garnered considerable attention due to its potential adaptability to saline conditions. Breeding and improving salt-tolerant varieties is a key strategy for the effective utilization of saline lands. Hence, it is important to conduct comprehensive research into the adaptability and salt tolerance mechanisms of *Brassica napus* in saline environments as well as to breed novel salt-tolerant varieties. This review summarizes the molecular mechanism of salt tolerance, physiological and phenotypic indexes, research strategies for the screening of salt-tolerant germplasm resources, and genetic engineering tools for salt stress in *Brassica napus*. It also introduces various agronomic strategies for applying exogenous substances to alleviate salt stress and provide technological tools and research directions for future research on salt tolerance in *Brassica napus*.

## 1. Introduction

Elevating soil salinization presents an environmental stressor that threatens the sustainability of crop production globally. Approximately 7% of the Earth’s land area is affected by soil salinity [1]. Due to land development and agricultural irrigation activities, the deterioration of this issue is on an upward trend [2]. As the global population continues to grow and food demand increases, the development of crop varieties resistant to salinity stress has become a pivotal research focus for enhancing global food security.

Rapeseed, a member of the Cruciferae family and *Brassica* genus, is the fifth major crop in China, following maize, rice, wheat, and soybeans. It encompasses a variety of species such as *Brassica napus*, *Brassica rapa*, and *Brassica juncea*. Among them, *Brassica napus* constitutes over 90% of China’s rapeseed cultivation area, and its oil contributes to more than 55% of the region’s total consumption of vegetable oils, making it a primary source of edible oil [3]. *Brassica napus* has an important economic value and is notable for its remarkable adaptability to adverse environmental conditions, particularly saline soils [4]. The need to enhance crop resilience against such stresses has led to a broad interest in understanding and improving *Brassica napus* salt tolerance, making it a subject of paramount research importance.

With the rapid development of molecular biology and genetic engineering, significant progress has been made in exploring the mechanisms behind salt tolerance in *Brassica napus*. Despite these advancements offering promising insights and methodologies for the development of salt-tolerant *Brassica napus* varieties, the complexity of the salt stress response and the constraints of current breeding technologies remain formidable challenges. The biological mechanisms of salt tolerance in staple crops like rice and maize have been extensively studied, leading to the isolation and cloning of key genes regulating plant salt tolerance. Conversely, the research on *Brassica napus* has predominantly concentrated on identifying physiological traits, mapping quantitative trait loci (QTLs), and functionally validating salt tolerance genes [5]. The molecular mechanisms and comprehensive regulatory networks governing salt tolerance in *Brassica napus* are still poorly understood. Therefore, exploring exceptional salt-tolerant germplasms, elucidating the roles of tolerance genes, deciphering their regulatory networks, and fostering the development of new germplasms with enhanced salt tolerance are crucial for advancing science and ensuring national food and oil security.

This review aims to provide a detailed overview of the current research on salt tolerance in *Brassica napus*, encompassing physiological and molecular mechanisms, genetic engineering approaches, and the prospects for breeding salt-tolerant varieties. By thoroughly mapping out the landscape of this critical research area, this article intends to guide future research directions and lay a foundation for ongoing and future efforts in plant science, thereby contributing significantly to the advancement of knowledge in this vital field.

## 2. Molecular Mechanism of Salt Stress on *Brassica napus*

The impact of salt stress on plants manifests through various mechanisms, including osmoregulation, ion homeostasis, and reactive-oxygen-species-scavenging systems. Short-term exposure to salt stress results in osmotic stress for plants. Elevated soil salt levels lead to an increased water potential, thereby hindering the short-term water absorption ability of plants [6]. This decrease in intracellular turgor pressure leads to reduced cellular activity, hindering cellular expansion and division, ultimately impeding normal plant growth and development. Concurrently, as the plant experiences water loss, leaf expansion becomes restricted and stomatal closure occurs on the leaf surface. This has an adverse effect on photosynthesis, and in severe cases, these physiological changes can ultimately lead to wilting and plant mortality [7]. Osmoregulation is a crucial mechanism that plays a pivotal role in the life cycle of plants under salt stress. Plants synthesize various osmotic and compatible solutes to mitigate the adverse effects caused by salt stress [8]. The abscisic acid (ABA) signaling pathway significantly contributes to the osmoregulatory responses induced by salt stress [9]. The accumulation of ABA is prompted by the augmentation of initial signaling involving calcium ions (Ca^2+^) and reactive oxygen species (ROS) prompts [10]. At the core of the ABA signaling pathway lies sucrose non-fermenting 1-related protein kinase (SnRK), a Ser/Thr protein kinase that is ubiquitously expressed in plants. When early salt stress signals are perceived by plants, the upstream kinases of the ABA signaling pathway, specifically B2, B3, and B4 Raf-like kinases, undergo rapid activation. These activated kinases then bind to SnRK2s, initiating a protein kinase cascade. SnRK2s, a upon activation, exhibit the capacity to phosphorylate positively regulate ABA-responsive element-binding protein (AREB) or ABA-binding factor (ABF) transcription factors [11]. Subsequently, this phosphorylation event serves as a regulatory mechanism for the modulation of plant stomatal aperture in response to salt stress [12]. Luo et al. [13] found in *Brassica napus* that the BnSIP1-1 (6b interacting protein 1) protein specifically targets the nucleus, and its expression can be induced by ABA and different stresses. Under osmotic pressure, salt, and ABA treatment, overexpression of *BnSIP1-1* improved seed germination. In addition, *BnSIP1-1* reduced the susceptibility of transgenic seedlings to osmotic pressure and ABA treatment, verifying that *BnSIP1-1* plays different regulatory mechanisms specifically in the osmotic and salt response pathways during the seedling stage [13].

The consequences of prolonged salt stress on plants are typically characterized by ion toxicity. Ion toxicity occurs when plants are subjected to elevated salt stress, leading to excessive uptake of salt ions, thereby disrupting the ionic balance in the plant [14]. This phenomenon results in nutrient deficiency, decelerated growth, suppression of photosynthesis, diminished organic matter accumulation, hastened senescence, and plant mortality [15]. The salt oversensitivity signaling pathway (SOS signaling pathway) constitutes a crucial regulatory mechanism governing ion homeostasis in plants. This pathway consists of three essential proteins: SOS1, SOS2, and SOS3. SOS1 functions as a Na^+^/H^+^ transporter located in the plasma membrane, SOS2 is a threonine protein kinase, and SOS3 serves as a Ca^2+^-binding protein [16]. In response to salt stress, plants exhibit an elevation in early-signal Ca^2+^ concentration. Subsequently, Ca^2+^ activates with SOS3, forming the SOS3-SOS2 complex, which activates SOS2. The SOS2 protein kinase then phosphorylates SOS1, initiating a cascade reaction. This sequence of events culminates in the efflux of Na^+^ from the plasma membrane via the SOS1-encoded Na^+^/H^+^ transporter protein, effectively reducing the intracellular Na^+^ concentration [17]. Moreover, SCaBP8 functions as a sensor for external Ca^2+^ signals, resulting in the formation of a complex with SOS2 kinase. This complex activates SOS1, contributing to the regulatory response under salt stress conditions [18]. This mechanism is crucial for maintaining intracellular ion homeostasis, thereby mitigating the deleterious effects of salt stress on plants. A lot of transporter proteins, including reverse transporter proteins, are involved in Na^+^/K^+^ homeostasis in the downstream of the signaling pathway that is responsive to salt stress. These proteins are primarily classified into three groups. The first group comprises Na^+^/H^+^ reverse transporter proteins belonging to the sodium/hydrogen exchanger (NHX) family, of which the unique structural feature of plant cells is the presence of vacuoles. Most of the Na^+^/H^+^ antiporters are localized in the vacuole and are involved in sequestration of Na^+^ within the vacuole under stressful conditions. These proteins play a key role in Na^+^ transport, facilitating the intracellular exocytosis of Na^+^ and vesicular Na^+^ storage [19]. Studies on the members of the Na^+^/H^+^ reverse transporter protein (NHX) family reveal their potential role in regulating resistance to salt stress in *Brassica napus*. Through bioinformatics and transcriptomic characterization, researchers identified that an overexpression of *BnaA05.NHX2* exhibited significantly higher salt tolerance than that of wild types [20]. The second category consists of the high-affinity K^+^ (HKT) gene family, which regulates the transport of Na^+^ and maintains the balance between Na^+^ and K^+^ during plant growth, development, and response to abiotic stress [21]. The researchers found that the plasma-membrane-localized *BnaC2.HKT1;1* is a central factor regulating Na^+^ unloading in root xylems [22]. The third category comprises HAK-type Na^+^-selective ion transport proteins, which play a role in mediating Na^+^ transport as well as facilitating K^+^ uptake and translocation in plant rhizomes [23]. There are 40 *BnaHAKs* genes in *Brassica napus*, which respond to nutritional stress such as low potassium and low salt. *BnaA7.HAK5* is located on the plasma membrane and is significantly upregulated under low potassium conditions. It is clear that *BnaA7.HAK5* plays an important role in the high-affinity absorption and transport of potassium ions in *Brassica napus* [24].

In response to salt stress, plants produce substantial quantities of ROS from various sources, including chloroplasts, mitochondria, and peroxisomes. These ROS include the superoxide anion (O^2−^), hydroxyl radical (OH^−^), singlet, and hydrogen peroxide (H_2_O_2_) [25]. While small quantities of ROS can function as signaling molecules, indicating abiotic stress in the plant organism, excessive accumulation can result in oxidative damage. This can disrupt cellular structure, hinder plant growth and development, harm biomolecules, and potentially induce programmed cell death [26]. To combat this, plants employ two primary ROS-scavenging mechanisms: enzymatic and non-enzymatic. Enzymatic scavenging relies on antioxidant enzymes like superoxide dismutase (SOD), peroxidase (POD), catalase (CAT), ascorbate peroxidase (APX), glutathione peroxidase (GPX), and glutathione S-transferase (GST). For example, SOD converts O^2−^ into H_2_O_2_ and O_2_, thereby reducing ROS toxicity [27]. Researchers conducted a comprehensive survey of the POD gene family in *Brassica napus*, identifying a total of 109 POD genes on 19 chromosomes. The *BnPODs* were found to be differentially expressed in different tissues, with higher expression levels observed in roots compared to other tissues. Furthermore, these genes exhibited varying expression levels under abiotic stress by using RT-qPCR [28]. Regarding the CAT gene family, a recent study identified 14 *BnCATs* in *Brassica napus*, which are closely associated with organelles, ROS response, stimulus response, stress response, and antioxidant enzyme activity. Specifically, *BnCAT1*-*BnCAT3* and *BnCAT11*-*BnCAT13* were significantly upregulated under different tissues and stress conditions such as low temperature, salinity, ABA, and gibberellin (GA) treatments, indicating their potential importance in plant stress response [29]. On the other hand, non-enzymatic scavenging involves the removal of ROS through compounds such as ascorbic acid (VC), reduced glutathione (GSH), α-tocopherol (VE), proline (Pro), alkaloids (carotenoids), and flavonoids. Through pot experiments, the differences in salt tolerance between two *Brassica napus* varieties (Shiralec and Dunkeld) were evaluated using the changes in MDA and H_2_O_2_ content with salt concentration treatment. It was found that the Shiralec has lower MDA and H_2_O_2_ content, indicating that its salt resistance is greater than that of the Dunkeld [30]. Together, these mechanisms help plants maintain ROS homeostasis and mitigate the damaging effects of salt stress [31].

## 3. Phenotypic and Physiological Indices of *Brassica napus* under Salt Stress

Phenotypic and physiological indicators are often relied upon in evaluating salt tolerance in *Brassica napus*. Screening salt-tolerant materials by measuring the amount of change in these indicators under salt stress is one of the strategies for salt tolerance research in *Brassica napus*.

### 3.1. Phenotypic Indices

The effect of salt stress on phenotypic development of *Brassica napus* is evident in various growth parameters. These are typically classified by fertility stage into germination, seedling, and maturity indicators, including seed germination, seedling root length, seedling height, stage biomass, survival rate, and biomass [32]. During the germination period, researchers usually determine the germination rate and germination potential of *Brassica napus*, along with other related indexes. For instance, Jiang et al. [33] treated 16 varieties of *Brassica napus* with salt stress using different salt concentration gradients (80, 160, and 240 mM). They found that the germination rate and germination potential of each variety were negatively correlated with the increase in salt concentration [33]. However, germination potential and germination rate were not always negatively correlated with salt concentration. Different salt concentrations (0, 25, 50, and 100 mM) were utilized by Fang et al. [34] to treat the germination stage of “yangyou 9”. It was found that 25 mM NaCl treatment promoted seed germination of “yangyou 9”, while 50 mM and 100 mM NaCl treatments inhibited seed germination of “yangyou 9” [34]. Long et al. [35] conducted a study in which fifteen *Brassica napus* inbred lines with diverse genetic backgrounds were exposed to varying levels of salt stress. Six traits, including germination rate, germination potential, root length, radicle length, fresh weight of a single plant, and fresh weight on the ground were evaluated at the germination stage. The findings revealed that the relative germination rate of *Brassica napus* followed a change in the Boltzmann curve as salt concentration increased. The optimal concentration for distinguishing *Brassica napus* was 214 mmol/L NaCl, which was the most appropriate concentration for identifying *Brassica napus* lines. At this concentration, root length and stem length were the most affected, followed by germination rate, germination potential, and total fresh weight. Aboveground fresh weight demonstrated the least susceptibility among these six traits. Furthermore, a significant positive correlation was observed between the relative values of these traits. Root length and stem length can be used as early evaluation indexes for salt tolerance in *Brassica napus* [35]. At the seedling stage, researchers usually choose indicators such as dry and fresh weight to determine the salt tolerance of *Brassica napus*. Researchers compared the performances of the three traits (embryonic axle length, total fresh weight, and aboveground fresh weight) of each variety in the presence of salt stress. At 80 mM NaCl, these traits were higher than that of the control, indicating that salt treatment promoted plant growth. However, at 160 and 240 mM NaCl, the values of all traits were significantly lower than those of the control group, suggesting that the salt treatment inhibited plant growth [33]. In addition to the determination of morphological indexes at the germination and seedling stages, physiological indexes at maturity are also important factors in determining the yield of *Brassica napus*. Wang et al. [36] examined the growth process, nitrogen and carbon characteristics, photosynthetic performance, biomass accumulation, and seed yield of *Brassica napus* under low and high soil salt ion concentration conditions. The findings indicated that the high-salt soil environment led to a delay of 4–5 days in the early stage and 6–8 days in the maturity stage, compared to the low-salt soil environment. In addition, N and C accumulation as well as the C/N ratio were reduced at both growth stages under high-salt soil conditions. It was suggested that salt stress disrupted the balance between carbon assimilation and nitrogen assimilation with a greater impact on carbon assimilation. Despite an increase in nitrogen content, the high-salt soil treatment resulted in a decrease in photosynthesis rate during the pre-flowering stage and a reduction in leaf area index at the onset of flowering. Additionally, N transport efficiency, particularly in the stem, and N utilization efficiency were diminished under high-salt soil conditions. Collectively, these adverse effects of high-salt soil contributed to lower biomass accumulation and seed yield [36].

### 3.2. Physiological Indices

The use of physiological indicators for screening salt-tolerant *Brassica napus* is usually based on three physiological indicators: osmoregulation, ion balance, and scavenging of reactive oxygen species. For osmotic stress, under low salt stress, the water potential of *Brassica napus* leaves decreased, and there was a decreasing trend in the soluble sugar content of most materials. However, under high salt stress, the water potential and the soluble sugar content of *Brassica napus* leaves significantly decreased. Betaine content tended to increase with increasing sodium chloride concentration [37,38]. In terms of oxidative stress, SOD, CAT, and APX activities showed an initial increase followed by a decrease, while GPX activity generally increased. Under low salt stress, the MDA content of *Brassica napus* leaves decreased significantly compared to CK. However, under high salt stress, the MDA content increased significantly [37,39]. In addition to the aforementioned factors, plants under salt stress exhibit excessive uptake of Na^+^, leading to secondary stresses such as nutrient mineral (K^+^) deficiencies and oxidative stresses [40]. The researchers stated that the ability of *Brassica napus* to accumulate sodium under salinity conditions is one of the main criteria for salt tolerance and observed higher K^+^ content in the shoots of salt-tolerant materials compared to sensitive materials at varying salinities [41]. Long et al. [42] conducted a comparison of Na^+^ and K^+^ contents in salt-tolerant and salt-sensitive materials, revealing slower increases in aboveground and belowground Na^+^ content and reduced loss of K^+^ in the former.

In summary, phenotypic studies on salt tolerance in *Brassica napus* can be divided into three stages according to the reproductive period: germination, seedling, and maturity. The assessment of germination rate, dry and fresh weight, and survival rate can be used as a reference for salt tolerance studies. Among them, it was observed that the germination potential, germination rate, relative shoot length, and relative root length initially increased and then decreased with the increase in salt concentration. This suggests that low salt concentration will promote seed germination and growth, thereby exerting a positive influence on its overall development. The physiological indicators of salt tolerance in *Brassica napus*, including soluble sugar, betaine, MDA, SOD, CAT, and the content of Na^+^ and K^+^, can likewise provide a reference for screening salt-tolerant accessions. Salt stress directly induced water loss and wilting along with excessive Na^+^ uptake and accumulation, causing osmotic stress and ionic imbalance in *Brassica napus*. The large accumulation of betaine and soluble sugar helps maintain osmotic potential balance to a certain extent and enhances the ability to tolerate salt stress. Furthermore, the elevation in SOD activities and the subsequent gradual increase in MDA content indicate that the elevated antioxidant enzyme activities mitigate damage caused by excess ROS to the cellular membrane system. Similarly, increased Na^+^ content in leaves and roots decreased K^+^ content in leaves and roots along with an altered K^+^/Na^+^ ratio result in ion imbalance within *Brassica napus*.

## 4. Research Strategy on Salt Tolerance of *Brassica napus*

As a moderately salt-tolerant crop, *Brassica napus* boasts a substantial foundation for enhancing its resilience to saline conditions, thus serving as a paradigm for driving the development of saline agriculture nationwide. This inherent resistance provides a solid foundation for improving its salt tolerance, making it easier to grow in highly saline areas. Despite its great potential, a comprehensive and systematic approach to improve salt tolerance in *Brassica napus* remains an unexplored territory.

### 4.1. Screening and Evaluation of Salt-Tolerant Germplasm

Plant salt tolerance is a complex trait that manifests through the modulation of diverse physiological and biochemical processes when confronted with salt stress. As a result, assessing salt tolerance and screening for superior germplasm resources are reliant on evaluating pertinent physiological and biochemical indicators. The impact of salt stress on plant morphology encompasses several observable alterations, including compromised root establishment, leaf curling and chlorosis, diminished tiller count per plant, reduced biomass, decreased plant height, and a decline in the 1000-seed weight [43,44]. Collectively, these effects culminate in a lowered harvest index and diminished seed yield [43,45,46]. Therefore, assessing salt tolerance and screening for superior salt-tolerant materials have become an important part of salt tolerance research in *Brassica napus*. Two main types of data collection methods are used: firstly, morphological indicators, which assess salt tolerance based on plant survival and biomass, providing a more intuitive and visible screening method and, secondly, physiological and biochemical factors, including measurements of chlorophyll content, cell membrane permeability, proline, soluble sugar, and soluble proteins. Alternatively, assessments incorporate a combination of both data types. Given that salt tolerance in *Brassica napus* is a polygenic quantitative trait, its inheritance mechanism is intricate, exhibiting significant variation across different growth and developmental stages with limited inter-stage correlation. During the preliminary assessment of salt tolerance traits, researchers often concentrate on the germination and seedling stages to gauge salt tolerance. For instance, Li et al. [47] conducted a comprehensive evaluation of *Brassica napus* germplasms’ salt tolerance through correlation, principal component, affiliation function, and cluster analyses in assessing *Brassica napus* salt tolerance during the germination period. By determining the germination potential, germination rate, root length, and shoot length of *Brassica napus*, they classified 146 *Brassica napus* into four types: salt-tolerant, moderately salt-tolerant, lowly salt-tolerant, and salt-insensitive materials [47]. Zhang et al. [48] analyzed morphological characteristics such as cotyledon length, hypocotyl length, root length, and stem thickness to determine the degree of *Brassica napus* salt tolerance at different salt concentrations. Wan et al. [49] utilized hydroponics to determine the salt tolerance of different *Brassica napus* varieties under different salt concentrations. This assessment included the germination potential, germination index, and salt tolerance of 11 agronomic traits at the seedling stage. These traits include the number of root hairs, radicle length, plant height, number of stems, stem thickness, vigor index, root fresh weight, root dry weight, and root volume. The composite salt tolerance values were used to assess the salt tolerance of the varieties [49]. These parameters are crucial for the comprehensive evaluation of salt tolerance in *Brassica napus* and are essential for studying this trait in the crop.

The acquisition of phenotypic data for salt tolerance relies on various sensors, enabling high-throughput plant phenotyping of morphological and physiological traits indicative of salt tolerance in real-world settings. These sensors include RGB imaging, spectral reflectance sensing, fluorescence imaging, thermal sensors, light detectors, laser, and ultrasound sensors. The extensive utilization of these sensors offers tremendous potential for high-throughput phenotyping of salt-tolerance-related traits in plants [50]. High-throughput phenotyping was employed in the investigation of salt tolerance in *Brassica napus* to identify plants with high repeatability and genetic significance under salt treatment. For example, researchers have obtained a map of 928 traits associated with salt stress response through stringent filtering criteria [51]. These traits were subsequently employed as biomarkers for predicting traditional traits related to salt stress, thereby facilitating the dissection of genetic base in *Brassica napus*.

### 4.2. Mining Salt Tolerance Genes by Genetics and Genomics in Brassica napus

#### 4.2.1. GWAS and QTL

In the study of the molecular mechanism of salt tolerance in *Brassica napus*, researchers have used gene cloning and other means to verify the effects of single or several salt tolerance genes. With the deepening understanding of the functional genome of *Brassica napus*, the rapid development of gene sequencing technology, and the use of the Genome-Wide Association Study (GWAS) analyses to identify genes or QTLs controlling salt tolerance throughout the whole life cycle of *Brassica napus*, there has been great facilitation in elucidating the salt tolerance mechanism and breeding salt-tolerant varieties. GWAS is one of the most common and productive methods for identifying genes in orthogenesis [52]. The association between adversity and plant genomic variation can be investigated through linkage mapping of inbred populations to locate relevant genes or by employing GWAS [53]. Researchers have evaluated the association of four seedling traits in 228 *Brassica napus* materials under salt stress using GWAS. They obtained 201,817 SNPs and identified 142 SNPs strongly related to salt tolerance, distributed across all chromosomal groups of *Brassica napus*. Among them, 78 SNPs were located on the C genome, and 64 SNPs were located on the A genome, respectively [54]. Zhang et al. [55] collected phenotypic data from 505 *Brassica napus* materials at the germination stage and seedling stage under 150 or 215 mM NaCl. Through a GWAS analysis, they investigated 16 salt tolerance coefficients, mapped 31 salt-stress-related genes, and detected 177 and 228 QTLs and candidate genes related to salt stress tolerance at the germination stage and seedling stage, respectively. Additionally, they enhanced the sensitivity to salt stress at the germination stage through the overexpression of two of the candidate genes, *BnCKX5* and *BnERF3* [55]. These findings highlight the potential of utilizing GWAS in conjunction with genotypic and phenotypic data to identify a rich pool of candidate genes for investigating the molecular mechanisms underlying salt stress adaptation in *Brassica napus*.

#### 4.2.2. Transcriptomics

Transcriptomics is one of the key research methods in the study of salt tolerance genotypes in *Brassica napus*. According to Hafiza Saima et al. [56], transcriptome analysis of the salt-sensitive variety Oscar revealed that salt stress downregulated differentially expressed genes (DEGs) associated with hormone signaling pathways, photosynthesis, and transcription factors, while upregulating DEGs related to amino acid biosynthesis and ion transport. These were combined with the fact that the Oscar variety was the worst at distinguishing between Na^+^ and K^+^ uptake and accumulation in the leaves and had a poorer antioxidant capacity for scavenging ROS. These findings suggest that the sensitivity of variety Oscar to salt stress is primarily attributed to inadequate control of ionic homeostasis, resulting in oxidative stress and diminished photosynthetic efficiency [56]. The researchers conducted a seven-day potting experiment of ZS11 with NaCl treatments (0, 100, and 200 mM) and 0.18 μM GR24 treatment, followed by a transcriptome analysis of roots and shoots. A total of 342 common DEGs and 166 specific DEGs were identified in the roots and shoots under the GR24 treatment. Notably, there was a significantly higher number of DEGs in roots compared to shoots. Quantitative PCR validation confirmed that stress relief was mainly related to the expression of genes for tryptophan metabolism, phytohormone signaling, and photosynthesis [57]. Currently, transcriptomics has also been applied in various plant salt tolerance studies, leading to the discovery of salt-tolerant transcription factors, such as bZIP, DREB, WRKY, MYB, and NAC, among others, through transcriptomic analyses [58]. Shu et al. [59] found that four transcription factors (*BnCAT2*, *BnWRKY40*, *BnHSFA2*, and *BnABF3*) were in leaves under salt stress, with peak expression at 24 h in *Brassica napus*. This was determined through a comparison of DEGs between the experimental and control groups in GO enrichment analysis. They also analyzed the enrichment of DEGs of other pathways such as ABA and MAPK signaling pathways, identifying numerous genes related to salt tolerance including *BnAUX* and *BnPYL*. Additionally, GO and KEGG annotation and clustering analyses were performed on the DEGs, and four differentially expressed proteins were identified: catalase-3, cysteine, HSP90, and P450-97A3 [59].

#### 4.2.3. Genetic Modification

Transgenic technology occupies a crucial role in the study of abiotic stress, with experts suggesting that genetically modified drought- and salt-tolerant plants can be used to economically utilize wastelands with excessive salt content and insufficient water [60]. For example, *Brassica napus* yield was increased by 2.34% under saline conditions by introducing the vesicular Na^+^/H^+^ anti-transporter gene *AtNHX1* [61]. Additionally, researchers have also utilized transgenic *Brassica napus* containing the constitutive gene of *YHem1* to enhance 5-ALA biosynthesis and investigate salt stress response. The findings demonstrated an increase in transgenic *Brassica napus* yield during long-term potting experiments as well as greater salt tolerance compared to wild types through measurements of various physiological indicators during short-term experiments [62]. These examples demonstrate the positive significance of transgenes in studying salt tolerance in *Brassica napus*.

Clustered Regularly Interspaced Short Palindromic Repeats-Cas (CRISPR/Cas)- based gene editing tools are characterized by simplicity, ease of use, adaptability, flexibility, and broad applicability. The system has great potential for breeding crop varieties that are more tolerant to abiotic stresses. Through the utilization of CRISPR/Cas9, researchers can target specific genes involved in stress response pathways to improve the ability of plants to withstand unfavorable environmental conditions. Abiotic stress tolerance is mainly controlled by QTLs in multiple genes [63]. CRISPR/Cas9-based genome editing technology allows for more precise genomic targeting and when the stress tolerance of a variety is determined by genes with variations in multiple SNPs. Advanced CRISPR tools can be employed to modify the SNPs and produce functional proteins that enhance the traits of the variety, thus avoiding time-consuming plant breeding procedures involving hybridization and selection [64]. CRISPR/Cas technology has been successfully applied in a variety of plant species [65] and has become an important tool for analyzing gene functions and molecular mechanisms in *Brassica napus*, contributing to the creation of *Brassica napus* germplasm resources and genetic improvements [66,67,68]. Based on the above studies, research on the molecular mechanism of salt tolerance in *Brassica napus* has only developed to the excavation of some genes related to salt stress, and there is no in-depth research on the regulatory network. While ion transporter proteins have always been the hotspot for studying the mechanism of salt tolerance in plants, there remains a paucity of studies on the excavation of ion transporter proteins in *Brassica napus*. In the future, a deeper understanding of the salt tolerance regulatory network in *Brassica napus* can be achieved by studying ionic transporter proteins. Therefore, future research should focus on mining the mechanism of salt tolerance and applying the research results to agricultural production practice.

#### 4.2.4. Pan-Genomics

Gene expression in plants plays a critical role in responding to salinity stress by activating or inhibiting specific genes. However, our understanding of the genetic variations that regulate salt stress gene expression is still limited. The pan-genome, which encompasses the entire genome library of a specific phylogenetic branch and encodes all potential lifestyles carried out by its organism, has been instrumental in revealing how genetic variations impact the dynamic changes in gene expression under salinity stress [69]. This technology has been effectively utilized in salt tolerance research of other crops; for example, researchers have identified 22,345 and 27,610 QTLs associated with the expression of 7787 and 936 genes under normal and salt stress conditions in rice, respectively [70]. Based on GWAS, researchers quickly identified the potential candidate gene *STG5*, which is necessary for maintaining Na^+^/K^+^ homeostasis by directly regulating the transcription of multiple members of the *OsHKT* gene family [70]. Currently, the pan-genome has also been widely used in the research of *Brassica napus* (Table 1). For example, researchers reported the sequencing, de novo assembly, and annotation of eight *Brassica napus* materials in a whole-genome association study (PAV-GWAS) that directly determined the causal structural changes in silique length, seed weight, and flowering time in a nested association mapping population with ZS11 as a donor [71]. However, in the study of salt tolerance in *Brassica napus*, no scholars have used pan-genome mining to identify key salt tolerance genes in *Brassica napus*. Therefore, assembling salt-tolerant germplasms of *Brassica napus* using the pan-genome to explore key salt-tolerant candidate genes may provide more key information for salt-tolerant research in *Brassica napus.*

## 5. Exogenous Substances Promote Growth under Salt Stress in *Brassica napus*

There are over 50 exogenous substances used in plants to alleviate salt stress, and they are classified into seven categories: regulation of ionic balance and pH, induction and synthesis of osmotic regulators, induction of antioxidant enzymes, hormonal regulation, induction of gene expression and signaling, improvement in photochemical systems, and microbial regulatory mechanisms (Table 2) [77]. In *Brassica napus*, many exogenous substances have also been reported to alleviate the effects of salt stress. The application of exogenous substances provides another way of thought in the study of salt tolerance [78].

## 6. Conclusions

**Figure 1:**
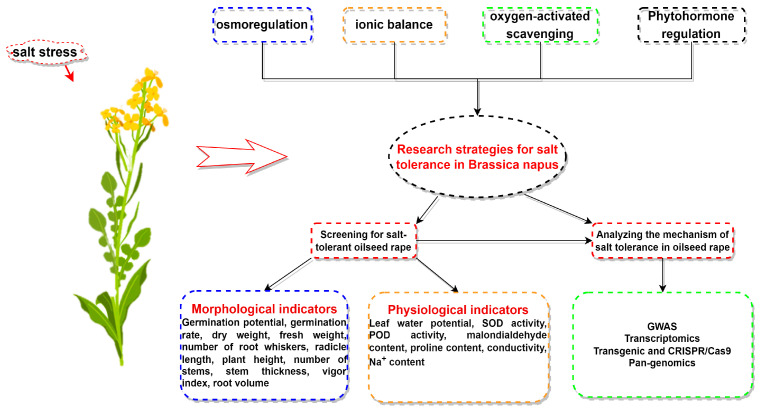
A series of physiological and biochemical responses occur in *Brassica napus* under a high-salt environment, including osmoregulation, ion homeostasis, scavenging of reactive oxygen species, and phytohormone regulation. When researchers study salt stress in *Brassica napus*, they usually screen and evaluate salt-tolerant and analyze the molecular mechanism of salt tolerance in rapeseed. When screening and evaluating salt-tolerant accessions, the morphological and physiological indexes are usually taken into consideration, among which the phenotypic indexes include germination potential, germination rate, dry weight, fresh weight, number of root whiskers, radicle length, plant height, number of stems, stem thickness, vitality index, and root volume, etc. The physiological indexes include leaf water potential, SOD activity, POD activity, malondialdehyde content, proline content, electrical conductivity, and Na^+^ content. In resolving the molecular mechanism of salt tolerance in *Brassica napus*, GWAS, transcriptomics, transgenes, CRISPR/Cas9, and pan-genomics are mostly used.

Plant coping strategies under salt stress include osmoregulation and ion homeostasis regulation (Figure 1). ABA signaling pathways and osmoregulatory substances participate in osmotic stress regulation. Meanwhile, the SOS pathway and downstream proteins governed by Na^+^- and K^+^-transporting proteins have been extensively researched for maintaining ion homeostasis. In addition, the involvement of reactive-oxygen-species-scavenging system and phytohormones also contribute to the regulatory mechanism of salt stress. For the study of salt tolerance in *Brassica napus*, emphasis is placed on investigating phenotypic and physiological indicators of *Brassica napus* under salt stress, providing a basis for identifying salt-tolerant varieties. It is worth noting that the phenotypic and physiological indexes of *Brassica napus* are not proportional to the salt concentration. At low concentrations, salt promotes growth, while at high concentrations, it inhibits growth. Furthermore, a range of genetic engineering tools including GWAS transcriptome analyses, transgenic technology, CRISPR/Cas9 editing, and pan-genomic approaches have been employed to investigate key salt tolerance genes in *Brassica napus*. In the case of *Brassica napus* exposed to salt stress, researchers commonly utilize exogenous substances to alleviate damage.

## 7. Discussion

In the investigation of plant salt tolerance, two primary approaches are employed: (1) exploring natural genetic variations between salt-tolerant and sensitive materials and (2) breeding transgenic materials with novel genes or manipulating the expression levels of existing genes. Current salt tolerance research predominantly involves dissecting the genetic basis of salt-tolerant plants by integrating phenotypic identification with molecular technologies. The emphasis is on large-scale screening of salt-tolerant materials to unveil the genetic basis, salt-tolerant genes, and associated regulatory networks, thereby revealing the intricate genetic underpinnings of salt tolerance. While physiological studies in *Brassica napus* have primarily focused on the germination or seedling stage, fewer investigations have extended to salt tolerance throughout the entire reproductive stage. Therefore, to comprehensively enhance *Brassica napus* salt tolerance without compromising yield and quality, it is imperative to study its physiological characteristics during the maturity stage, yield variations, and the impact of saline cultivation on *Brassica napus* quality. Due to substantial variations in salt stress tolerance among various *Brassica napus*, there is a lack of standardization in research techniques for *Brassica napus* salt tolerance, emphasizing the necessity for further advancements in salt tolerance research, particularly in establishing a unified standard. Future research on salt tolerance in *Brassica napus* should be deepened in four key areas: (1) establishing a technical system for the identification and evaluation of salt tolerance traits, involving the development of a standardized, high-throughput, digital salt tolerance trait identification and evaluation technology system; (2) taking advantage of multi-omics technology to comprehensively analyze the biological mechanisms underlying salt tolerance and to discover key genes; (3) creating superior salt-tolerant germplasm resources by identifying allelic variants of key salt tolerance genes for molecular breeding, which involves establishing an efficient molecular breeding technology system and applying newly discovered salt tolerance genes in agricultural production to cultivate new salt-tolerant varieties; and (4) promoting the adaptation of *Brassica napus* to high-salt soils by external interventions in physiology and biochemistry. Given that *Brassica napus* is the primary source of domestically produced vegetable oil in China, expanding its planting area and increasing production output can alleviate food and oil supply shortages. However, decreasing arable land due to climate and environmental changes poses a constraint. It has become critical to utilize the broad adaptability of rapeseed for growth in high-salt areas. Hence, identifying key regulatory genes for salt tolerance, elucidating its biological mechanisms, and creating salt-tolerant germplasm resources are of paramount importance for China’s grain and oil security.

## Figures and Tables

**Table 1:** Salt-tolerance-related genes in *Brassica napus*.

Gene Name	Gene ID	*Arabidopsis thaliana* Gene Number	Features	Function	Reference
*BnCKX5*	BnaA02G0088800ZS	AT5G21482	Encodes cytokinin dehydrogenase, which plays a role in maintaining cytokinin homeostasis and is considered a candidate gene for salt stress response.	unclear	[55]
*BnERF3*	BnaA06G0028500ZS	AT1G50640	Encodes an ethylene response transcription factor that plays an important role in signal transduction in many adversity stresses and is considered a candidate gene for salt stress response.	unclear	[55]
*BnNAC2* *BnNAC5*	BnaA07T0309700ZS BnaA10T0224100ZS	AT1G69490 AT5G13180	Induced by high salt, drought, and ABA and encodes a member of the NAC transcription factor gene family. It is expressed in the floral primordium and is highly regulated by AP3 and PI. Its expression is associated with leaf senescence.	negative	[72]
*BnPYL1-2* *BnPYL7-2*	BnaA06G0421700ZS BnaA03G0276800ZS	AT5G46790 AT4G01026	PYL is involved in the first step of ABA signaling, and some *BnPYL* genes are responsive to abiotic stresses such as salt and high temperature.	positive	[73]
*BnRH6*			*BnRH6* is targeted at the nucleus and cytoplasmic processing body (P-body), constitutively expressed throughout the lifespan, and induced by salt stress.	negative	[74]
*BnSWD1*			*BnSWD1* gene is expressed at high levels under salt stress conditions, which was upregulated after treatment with abscisic acid, salicylic acid, and methyl jasmonate.	unclear	[75]
*BnaA.TTG2.a.1*		AT2G37260	*BnaA.TTG2.a.1* reduces IAA levels by repressing the expression of IAA synthesis genes, thereby making overexpressing plants salt-sensitive.	negative	[76]

**Table 2:** Salt-tolerance-related exogenous substance in *Brassica napus*.

Exogenous Substances	Application Method	Optimum Concentration	Mechanism of Exogenous Substances	Reference
Serotonin	Hydroponically	200 µM	Serotonin increases the activity of catalase (CAT), superoxide dismutase (SOD), and peroxidase (POD), effectively activating the antioxidant enzyme system with the ability to scavenge reactive oxygen species, regulate osmotic pressure, and promote growth.	[79]
Melatonin	Hydroponically	30 µM	Low concentrations of exogenous melatonin increase the activities of antioxidant enzymes such as POD, CAT, and APX; promote the accumulation of soluble proteins, proline, and water-soluble glucan; promote root development; and increase the biomass of *Brassica napus* seedlings under salt stress.	[80]
Salicylic acid	Foliar application	0.02 M	SA treatment increases shoot fresh weight, root dry weight, chlorophyll a, chlorophyll b, superoxide dismutase, peroxidase, catalase, total soluble proteins, total soluble sugars, total phenolics, flavonoids, anthocyanins, endogenous ascorbic acid, glycine betaine, and total free proline.	[81]
ZnSO_4_ ZNP	Foliar application	75 mg/L 10 mg/L	Zinc application reduces MP%, MDA, and H_2_O_2_ concentrations. Increased accumulation of proline and total soluble carbohydrates, nitrogen, potassium, and phosphorus content in plant tissues decreased sodium and chloride content.	[82]
Isosteviol	Seed soaking	10^−9^~10^−8^ M	Isosteviol regulates the production of osmotic substances and ROS and reduces oxidative damage caused by salt stress in *Brassica napus* seedlings. Isosteviol also reduces Na^+^ uptake by seedling tissues, increases K^+^ content, and mitigates the damage caused by salt stress to plant seedlings.	[83]
bio-selenium nanoparticles	Pre-seed treatment	150 μM	Biological SeNPs enhance seed vigor, improve seedling growth and physicochemical properties, regulate Na^+^ and K^+^ uptake, and improve rapeseed growth.	[84]
Poly (γ-glutamic acid)	Hydroponically	20 mg/L	γ-PGA increases the resistance of *Brassica napus* seedlings to salt stress by activating the proline synthesis pathway and promoting proline accumulation.	[85]
Ellagic acid	Seed soaking	Applied based on salt concentration	Application of EA as a seed soak mitigates the effects of salinity and promotes plant growth.	[86]
Brassinosteroids	Hydroponically		Exogenous EBL impedes the development of NaCl-dependent lipid peroxidation and increases the osmotic potential of leaf cell contents. The protective effect of EBL under salt stress may be related to the ability of EBL to maintain intracellular ionic homeostasis regulating water status by its antioxidant action.	[87]
Nitrogen	Hydroponically		Nitrogen significantly affects the contents of MDA, proline, chlorophyll, and total nitrogen in the early stage of growth, but significantly affects the contents of leaf water and soluble sugar in the late stage of growth.	[88]
5-aminolevulinic acid	Foliar application	30 mg/L	ALA promotes increased levels of intermediates of the tetrapyrrole biosynthesis pathway, which promotes the accumulation of chlorophyll and heme, as well as enhances the accumulation of proline, thereby improving salt tolerance in *Brassica napus.*	[89,90]
Selemium	Foliar application	5 mg/L	Se applied alone or in combination with salt treatments significantly increases plant growth, plant yield, and photosynthetic pigment content and improves the quality of *Brassica napus* oil.	[91]
Hemin	Foliar application		Hematoxylin (HS) significantly improves root length, seedling height, stem diameter and accumulates more dry matter biomass in *Brassica napus* seedlings under NaCl stress. It improves photosynthetic efficiency; increases the activities of antioxidant enzymes such as superoxide dismutase (SOD), peroxidase (POD), and ascorbate peroxidase (APX); reduces electrolyte leakage (EL) and malondialdehyde (MDA) content; and mitigates oxidative membrane damage.	[92]
Silicon	Hydroponically		Silicon nutrition ameliorates the deleterious effects of salinity on the growth of *Brassica napus* plants by reducing the Na^+^ content of tissues and maintaining the integrity of root cell membranes.	[93]
Polyamines	Foliar application		Exogenous application of Spd regulates antioxidant enzyme activities, the polyamine pathway, and Calvin cycle enzyme-related genes to alleviate salt stress injury in plants.	[94]

## References

[1] Munns R., Tester M. (2008). Mechanisms of salinity tolerance. Annu. Rev. Plant Biol..

[2] Ismail A.M., Horie T. (2017). Genomics, Physiology, and molecular breeding approaches for improving salt tolerance. Annu. Rev. Plant Biol..

[3] Wang H. (2018). New-demand oriented oilseed rape industry developing strategy. Chin. J. Oil Crop Sci..

[4] Wang W., Ge Z., Yang H., Yin F., Huang T., Kuai J., Wang J., Wang B., Zhou G., Fu T. (2022). Daptation of feed crops to saline-alkali soil Stress and effect of improving saline-alkali soil. Acta Agron. Sin..

[5] Zhang Y., Zhang Q., Wang H., Tao S., Cao H., Shi Y., Bakirov A., Xu A., Huang Z. (2023). Discovery of common loci and candidate genes for controlling salt-alkali tolerance and yield-related traits in *Brassica napus* L.. Plant Cell Rep..

[6] Munns R., Passioura J.B., Colmer T.D., Byrt C.S. (2020). Osmotic adjustment and energy limitations to plant growth in saline soil. New Phytol..

[7] Xia D. (2020). Research progress and breeding of plant drought resistance physiology. Mod. Hortic..

[8] Adem G.D., Roy S.J., Zhou M., Bowman J.P., Shabala S. (2014). Evaluating contribution of ionic, osmotic and oxidative stress components towards salinity tolerance in barley. BMC Plant Biol..

[9] Yang W., Li N., Fan Y., Dong B., Song Z., Cao H., Du T., Liu T., Qi M., Niu L. (2021). Transcriptome analysis reveals abscisic acid enhancing drought resistance by regulating genes related to flavonoid metabolism in pigeon pea. Environ. Exp. Bot..

[10] Yu Z., Duan X., Luo L., Dai S., Ding Z., Xia G. (2020). How plant hormones mediate salt stress responses. Trends Plant Sci..

[11] Hasan M.M., Liu X.-D., Waseem M., Guang-Qian Y., Alabdallah N.M., Jahan M.S., Fang X.-W. (2022). ABA activated SnRK2 kinases: An emerging role in plant growth and physiology. Plant Signal. Behav..

[12] Amjad M., Akhtar J., Anwar-ul-Haq M., Yang A., Akhtar S.S., Jacobsen S.E. (2014). Integrating role of ethylene and ABA in tomato plants adaptation to salt stress. Sci. Hortic..

[13] Luo J., Tang S., Mei F., Peng X., Li J., Li X., Yan X., Zeng X., Liu F., Wu Y. (2017). *BnSIP1-1*, a trihelix family gene, mediates abiotic stress tolerance and ABA signaling in *Brassica napus*. Front. Plant Sci..

[14] Zhao S., Zhang Q., Liu M., Zhou H., Ma C., Wang P. (2021). Regulation of plant responses to salt stress. Int. J. Mol. Sci..

[15] Hong S., Yin M. (2008). Effects of Salt Stress on the growth and some physiological and biochemical indices of hybrid rice plantlets in Vitro. Hybrid. Rice.

[16] Yang Y., Guo Y. (2018). Unraveling salt stress signaling in plants: Salt stress signaling. J. Integr. Plant Biol..

[17] Köster P., Wallrad L., Edel K.H., Faisal M., Alatar A.A., Kudla J. (2019). The battle of two ions: Ca^2+^ signalling against Na^+^ stress. Plant Biol..

[18] Ma L., Ye J., Yang Y., Lin H., Yue L., Luo J., Long Y., Fu H., Liu X., Zhang Y. (2019). The SOS2-SCaBP8 complex generates and fine-tunes an *AtANN4*-dependent calcium signature under salt stress. Dev. Cell.

[19] Yarra R. (2019). The wheat NHX gene family: Potential role in improving salinity stress tolerance of plants. Plant Gene.

[20] Yue C., Han L., Sun S., Chen J., Feng Y., Huang J., Zhou T., Hua Y. (2024). Genome-wide identification of the cation/proton antiporter (CPA) gene family and functional characterization of the key member *BnaA05. NHX2* in allotetraploid rapeseed. Gene.

[21] Li H., Xu G., Yang C., Yang L., Liang Z. (2019). Genome-wide identification and expression analysis of HKT transcription factor under salt stress in nine plant species. Ecotoxicol. Environ. Saf..

[22] Zhou T., Yue C.-P., Liu Y., Zhang T.-Y., Huang J.-Y., Hua Y.-P. (2021). Multiomics reveal pivotal roles of sodium translocation and compartmentation in regulating salinity resistance in allotetraploid rapeseed. J. Exp. Bot..

[23] Yang Y., Guo Y. (2018). Elucidating the molecular mechanisms mediating plant salt-stress responses. New Phytol..

[24] Chen J., Song H., Zhou T., Yue C., Feng Y., Huang J., Hua Y. (2024). Identification of the core member of the HAKs family and primary analysis on their functions in allotetraploid rapeseed (*Brassica napus* L.). J. Plant Nutr. Fertil..

[25] Qi J., Song C.-P., Wang B., Zhou J., Kangasjärvi J., Zhu J.K., Gong Z. (2018). Reactive oxygen species signaling and stomatal movement in plant responses to drought stress and pathogen attack: ROS signaling and stomatal movement. J. Integr. Plant Biol..

[26] Halliwell B. (2006). Reactive species and antioxidants. Redox biology is a fundamental theme of aerobic life. Plant Physiol..

[27] Shahzad B., Rehman A., Tanveer M., Wang L., Park S.K., Ali A. (2022). Salt stress in brassica: Effects, tolerance mechanisms, and management. J. Plant Growth Regul..

[28] Shah O.U., Khan L.U., Basharat S., Zhou L., Ikram M., Peng J., Khan W.U., Liu P., Waseem M. (2024). Genome-Wide Investigation of Class III Peroxidase Genes in *Brassica napus* Reveals Their Responsiveness to Abiotic Stresses. Plants.

[29] Raza A., Su W., Gao A., Mehmood S.S., Hussain M.A., Nie W., Lv Y., Zou X., Zhang X. (2021). Catalase (CAT) Gene Family in Rapeseed (*Brassica napus* L.): Genome-Wide Analysis, Identification, and Expression Pattern in Response to Multiple Hormones and Abiotic Stress Conditions. Int. J. Mol. Sci..

[30] Rasheed R., Ashraf M.A., Parveen S., Iqbal M., Hussain I. (2014). Effect of salt stress on different growth and biochemical attributes in two canola (*Brassica napus* L.) cultivars. Commun. Soil. Sci. Plant Anal..

[31] Guo M., Liu J., Hou L., Zhang T., Liu H. (2021). Research progress on the production and clearance mechanism of reactive oxygen species in plants. Sci. Technol. Vis..

[32] Zhang X., Yang X., Jiao Z. (2018). Research progress of salt tolerance evaluation in plants and tolerance evaluation strategy. J. Biol..

[33] Jiang J., Zhang J., Yang L., Zhu J., Wang W., Lei L., Zhou X., Li Y. (2024). Effects of salt stress on germination of rapeseed seeds. Mol. Plant Breed..

[34] Fang Y., Li J., Jiang J., Geng Y., Wang J., Wang Y. (2017). Physiological and epigenetic analyses of *Brassica napus* seed germination in response to salt stress. Acta Physiol. Plant..

[35] Long W., Pu H., Zhang J., Qi C., Zhang X. (2013). Screening of *Brassica napus* for salinity tolerance at germination stage. Chin. J. Oil Crop Sci..

[36] Wang L., Zuo Q., Zheng J., You J., Yang G., Leng S. (2022). Salt stress decreases seed yield and postpones growth process of canola (*Brassica napus* L.) by changing nitrogen and carbon characters. Sci. Rep..

[37] Ding J., Huang Z., Zhang X., Lu H., Liu L., Xu A. (2014). Physiological effects on *Brassica napus* seedlings under NaCl stress. Acta Bot. Boreali-Occident. Sin..

[38] Yang Y., Zheng Q., Liu M., Guo S. (2012). Difference in sodium spatial distribution in the shoot of two canola cultivars under saline stress. Plant Cell Physiol..

[39] Zhang G., Kou M., Wang Y., Bai Y., Zhang J. (2014). The effect of physiological indices in two *Brassica napus* leaves under salt stress. J. Northwest Norm. Univ. (Nat. Sci.).

[40] Keshavarzian M., Toorchi M., Shakiba M.R. (2020). Sodium chloride salt tolerance evaluation and classification of spring rapeseed (*Brassica napus* L.). J. Crop Breed..

[41] Pak V.A., Nabipour M., Meskarbashee M. (2009). Effect of salt stress on chlorophyll content, fluorescence, Na and K ions content in rape plants (*Brassica napus* L.). Asian J. Agric. Res..

[42] Long W., Gao J., Hu M., Chen S., Zhang J., Qi C., Zhang X., Pu H. (2016). Cation accumulation characteristics of rapeseed under salt stress. Chin. J. Oil Crop Sci..

[43] Soltabayeva A., Ongaltay A., Omondi J.O., Srivastava S. (2021). Morphological, physiological and molecular markers for salt-stressed plants. Plants.

[44] Zuo Q., Liu J., Shan J., Zhou J., Wang L., Yang G., Leng S., Liu H. (2019). Carbon and nitrogen assimilation and partitioning in canola (*Brassica napus* L.) in saline environment. Commun. Soil. Sci. Plant Anal..

[45] Yadav S.P., Bharadwaj R., Nayak H., Mahto R., Singh R.K., Prasad S.K. (2019). Impact of salt stress on growth, productivity and physicochemical properties of plants: A Review. Int. J. Chem. Stud..

[46] Acosta-Motos J.R., Ortuño M.F., Bernal-Vicente A., Diaz-Vivancos P., Sanchez-Blanco M.J., Hernandez J.A. (2017). Plant responses to salt stress: Adaptive mechanisms. Agronomy.

[47] Li P., Yan J., Zhang H., Zhang Y., Tao S., Zhang Q., Aldiyar, Xu A., Huang Z. (2021). Screening and evaluation of salt tolerance for 146 *Brassica napus* germplasms at germination Stage. Acta Agric. Boreali-Occident. Sin..

[48] Zhang Y., Gao S., Zhang R., Xie K., Wang J. (2021). The effect of salt stress on the germination of different rapeseed seeds. Seed.

[49] Wan L., Ni Z., Sun H. (2019). Rapid Screening of salt tolerance of rapeseed by laboratory hydroponics method. J. Jinling Inst. Technol..

[50] Hu Y., Schmidhalter U. (2023). Opportunity and challenges of phenotyping plant salt tolerance. Trends Plant Sci..

[51] Zhang G., Zhou J., Peng Y., Tan Z., Zhang Y., Zhao H., Liu D., Liu X., Li L., Yu L. (2021). High-throughput phenotyping-based QTL mapping reveals the genetic architecture of the salt stress tolerance of *Brassica napus*. Plant Cell Environ..

[52] Du W., Ning L., Liu Y., Zhang S., Yang Y., Wang Q., Chao S., Yang H., Huang F., Cheng H. (2020). Identification of loci and candidate gene *GmSPX-RING1* responsible for phosphorus efficiency in soybean via genome-wide association analysis. BMC Genom..

[53] Nordborg M., Atwell S., Huang Y.S., Vilhjálmsson B.J., Willems G., Horton M., Li Y., Meng D., Platt A., Tarone A.M. (2010). Genome-wide association study of 107 phenotypes in Arabidopsis thaliana inbred lines. Nature.

[54] Wassan G.M., Khanzada H., Zhou Q., Mason A.S., Keerio A.A., Khanzada S., Solangi A.M., Faheem M., Fu D., He H. (2021). Identification of genetic variation for salt tolerance in *Brassica napus* using genome-wide association mapping. Mol. Genet. Genom..

[55] Zhang G., Zhou J., Peng Y., Tan Z., Li L., Yu L., Jin C., Fang S., Lu S., Guo L. (2022). Genome-wide association studies of salt tolerance at seed germination and seedling stages in *Brassica napus*. Front. Plant Sci..

[56] Gul H.S., Ulfat M., Zafar Z.U., Haider W., Ali Z., Manzoor H., Afzal S., Ashraf M., Athar H.-U.-R. (2022). Photosynthesis and salt exclusion are key physiological processes contributing to salt tolerance of canola (*Brassica napus* L.): Evidence from physiology and transcriptome analysis. Genes.

[57] Ma N., Hu C., Wan L., Hu Q., Xiong J., Zhang C. (2017). Strigolactones improve plant growth, photosynthesis, and alleviate oxidative stress under salinity in rapeseed (*Brassica napus* L.) by regulating gene expression. Front. Plant Sci..

[58] Baillo E.H., Kimotho R.N., Zhang Z., Xu P. (2019). Transcription factors associated with abiotic and biotic stress tolerance and their potential for crops improvement. Genes.

[59] Shu J., Ma X., Ma H., Huang Q., Zhang Y., Guan M., Guan C.F. (2022). Transcriptomic, proteomic, metabolomic, and functional genomic approaches of *Brassica napus* L. during salt stress. PLoS ONE.

[60] Ahmad P., Ashraf M., Younis M., Hu X., Kumar A., Akram N.A., Al-Qurainy F. (2012). Role of transgenic plants in agriculture and biopharming. Biotechnol. Adv..

[61] Zhang H.X., Hodson J.N., Williams J.P., Blumwald E. (2001). Engineering salt-tolerant Brassica plants: Characterization of yield and seed oil quality in transgenic plants with increased vacuolar sodium accumulation. Proc. Natl. Acad. Sci. USA.

[62] Sun X., Feng X., Li C., Zhang Z., Wang L. (2015). Study on salt tolerance with *YHem1* transgenic canola (*Brassica napus*). Physiol. Plant..

[63] Jha U.C., Bohra A., Nayyar H. (2020). Advances in “omics” approaches to tackle drought stress in grain legumes. Plant Breed..

[64] Kumar M., Prusty M.R., Pandey M.K., Singh P.K., Bohra A., Guo B., Varshney R.K. (2023). Application of CRISPR/Cas9-mediated gene editing for abiotic stress management in crop plants. Front. Plant Sci..

[65] Zhang D., Li Z., Li J.-F. (2016). Targeted gene manipulation in plants using the CRISPR/Cas technology. J. Genet. Genom..

[66] Cabral G.B., Ferreira J.L.d.P., Souza R.P.d., Cunha M.S., Luchs A., Figueiredo C.A., Brígido L.F.d.M. (2017). Simple protocol for population (Sanger) sequencing for Zika virus genomic regions. Memórias Inst. Oswaldo Cruz.

[67] Brookes G., Barfoot P. (2018). Environmental impacts of genetically modified (GM) crop use 1996–2016: Impacts on pesticide use and carbon emissions. GM Crops Food.

[68] Bernabé-Orts J.M., Casas-Rodrigo I., Minguet E.G., Landolfi V., Garcia-Carpintero V., Gianoglio S., Vázquez-Vilar M., Granell A., Orzaez D. (2019). Assessment of Cas12a-mediated gene editing efficiency in plants. Plant Biotechnol. J..

[69] Vernikos G., Medini D., Riley D.R., Tettelin H. (2015). Ten years of pan-genome analyses. Curr. Opin. Microbiol..

[70] Wei H., Wang X., Zhang Z., Yang L., Zhang Q., Li Y., He H., Chen D., Zhang B., Zheng C. (2024). Uncovering key salt-tolerant regulators through a combined eQTL and GWAS analysis using the super pan-genome in rice. Natl. Sci. Rev..

[71] Song J., Guan Z., Hu J., Guo C., Yang Z., Wang S., Liu D., Wang B., Lu S., Zhou R. (2020). Eight high-quality genomes reveal pan-genome architecture and ecotype differentiation of *Brassica napus*. Nat. Plants.

[72] Zhong H., Guo Q.Q., Chen L., Ren F., Wang Q.Q., Zheng Y., Li X.B. (2012). Two *Brassica napus* genes encoding NAC transcription factors are involved in response to high-salinity stress. Plant Cell Rep..

[73] Di F., Jian H., Wang T., Chen X., Ding Y., Du H., Lu K., Li J., Liu L. (2018). Genome-wide analysis of the PYL gene family and identification of PYL genes that respond to abiotic stress in *Brassica napus*. Genes.

[74] Zhang X., Song J., Wang L., Yang Z., Sun D. (2022). Identification of a DEAD-box RNA helicase BnRH6 reveals its involvement in salt stress response in rapeseed (*Brassica napus*). Int. J. Mol. Sci..

[75] Lee S., Lee J., Paek K.H., Kwon S.Y., Cho H.S., Kim S.J., Park J.M. (2010). A novel WD40 protein, *BnSWD1*, is involved in salt stress in *Brassica napus*. Plant Biotechnol. Rep..

[76] Li Q., Yin M., Li Y., Fan C., Yang Q., Wu J., Zhang C., Wang H., Zhou Y. (2015). Expression of *Brassica napus* TTG2, a regulator of trichome development, increases plant sensitivity to salt stress by suppressing the expression of auxin biosynthesis genes. J. Exp. Bot..

[77] Gao Q., Feng D., Liu J., Zhang J., Han Q. (2021). Main mechanisms and classification of exogenous substances alleviating plant salt stress. J. Plant Nutr. Fertil..

[78] Shah A.N., Tanveer M., Abbas A., Fahad S., Baloch M.S., Ahmad M.I., Saud S., Song Y. (2021). Targeting salt stress coping mechanisms for stress tolerance in Brassica: A research perspective. Plant Physiol. Biochem..

[79] Liu Y., Ding X., Lv Y., Cheng Y., Li C., Yan L., Tian S., Zou X. (2021). Exogenous serotonin improves salt tolerance in rapeseed *(Brassica napus* L.) seedlings. Agronomy.

[80] Liu Z., Cai J., Li J., Lu G., Li C., Fu G., Zhang X., Liu Q., Zou X., Cheng Y. (2018). Exogenous application of a low concentration of melatonin enhances salt tolerance in rapeseed (*Brassica napus* L.) seedlings. J. Integr. Agric..

[81] Ilyas M., Maqsood M.F., Shahbaz M., Zulfiqar U., Ahmad K., Naz N., Ali M.F., Ahmad M., Ali Q., Yong J.W.H. (2024). Alleviating salinity stress in canola (*Brassica napus* L.) through exogenous application of salicylic acid. BMC Plant Biol..

[82] Farouk S., Al-Amri S.M. (2019). Exogenous zinc forms counteract NaCl-induced damage by regulating the antioxidant system, osmotic adjustment substances, and ions in canola (*Brassica napus* L. cv. Pactol) plants. J. Soil. Sci. Plant Nutr..

[83] Xia W., Meng W., Peng Y., Qin Y., Zhang L., Zhu N. (2024). Effects of Exogenous Isosteviol on the Physiological Characteristics of *Brassica napus* Seedlings under Salt Stress. Plants.

[84] El-Badri A.M., Batool M., Mohamed I.A., Wang Z., Wang C., Tabl K.M., Khatab A., Kuai J., Wang J., Wang B. (2022). Mitigation of the salinity stress in rapeseed (*Brassica napus* L.) productivity by exogenous applications of bio-selenium nanoparticles during the early seedling stage. Environ. Pollut..

[85] Lei P., Xu Z., Liang J., Luo X., Zhang Y., Feng X., Xu H. (2016). Poly (γ-glutamic acid) enhanced tolerance to salt stress by promoting proline accumulation in *Brassica napus* L.. Plant Growth Regul..

[86] Khan A., Nazar S., Lang I., Nawaz H., Hussain M.A. (2017). Effect of ellagic acid on growth and physiology of canola (*Brassica napus* L.) under saline conditions. J. Plant Interact..

[87] Efimova M., Savchuk A., Hasan J., Litvinovskaya R., Khripach V., Kholodova V., Kuznetsov V.V. (2014). Physiological mechanisms of enhancing salt tolerance of *Brassica napus* plants with brassinosteroids. Russ. J. Plant Physiol..

[88] Tian T., Wang H., Wang J., Zhu Y., Shi X., Li W. (2021). Effects of nitrogen application on accumulation of organic osmotic regulating substances in forage rapeseed (*Brassica napus*) under salt stress. Acta Prataculturae Sin..

[89] Xiong J., Wang H., Tan X., Zhang C., Naeem M.S. (2018). 5-aminolevulinic acid improves salt tolerance mediated by regulation of tetrapyrrole and proline metabolism in *Brassica napus* L. seedlings under NaCl stress. Plant Physiol. Biochem..

[90] Naeem M.S., Warusawitharana H., Liu H., Liu D., Ahmad R., Waraich E.A., Xu L., Zhou W. (2012). 5-Aminolevulinic acid alleviates the salinity-induced changes in *Brassica napus* as revealed by the ultrastructural study of chloroplast. Plant Physiol. Biochem..

[91] Hashem H.A., Hassanein R.A., Bekheta M.A., El-Kady F.A. (2013). Protective role of selenium in canola (*Brassica napus* L.) plant subjected to salt stress. Egypt. J. Exp. Biol..

[92] Zhao H.-M., Zheng D.-F., Feng N.-J., Zhou G.-S., Khan A., Lu X.-T., Deng P., Zhou H., Du Y.-W. (2023). Regulatory effects of Hemin on prevention and rescue of salt stress in rapeseed (*Brassica napus* L.) seedlings. BMC Plant Biol..

[93] Hashemi A., Abdolzadeh A., Sadeghipour H.R. (2010). Beneficial effects of silicon nutrition in alleviating salinity stress in hydroponically grown canola, *Brassica napus* L., plants. Soil. Sci. Plant Nutr..

[94] ElSayed A.I., Mohamed A.H., Rafudeen M.S., Omar A.A., Awad M.F., Mansour E. (2022). Polyamines mitigate the destructive impacts of salinity stress by enhancing photosynthetic capacity, antioxidant defense system and upregulation of calvin cycle-related genes in rapeseed (*Brassica napus* L.). Saudi J. Biol. Sci..

